# Therapeutics

**Published:** 1877-03

**Authors:** 


### IV. Therapeutics.

1. *A Remedy for Whooping Cough.* Lasinski. (*Deutsche Med. Wochenschr.*, 1877, No. 2.)

In twenty-five cases of whooping cough the author has been so exceedingly successful with his topical medication that he has no hesitation in recommending it very warmly to the profession. His remedy is the following powder: ℞. Quin. Muriat., 1.0; Ac. Salicyl., 2.0; Sacch. Alb., Sod. Bicarbon. *aa*, 0.5 (1 gramme=15 grains. See Journal and Examiner, February, 1877, p. 172.) This powder is applied to the affected larynx by means of a laryngeal insufflator; the insufflations are made twice daily, and the above quantity of the remedy will last ten days. Consequently, at each application about 0.05 quinine and 0.1 salicylic acid are used. The small dose of the powder being put in the open end of the insufflator, the patient is told to put out his tongue and to take a deep inspiration. At this very moment the tube of the insufflator is quickly put into the mouth far enough to get its curved end behind the epiglottis, and the powder is blown into the larynx. Although the children naturally struggled, they could be managed by one person who had them on his lap and held their hands. Small children of course would not inspire just at the demand of the surgeon, who then had to wait and watch for the desired moment to insufflate the powder. But, for all this difficulty, the whole manipulation never occupied more than three minutes. When the powder actually was blown into the larynx it caused an attack of suffocation, so that this phenomenon may be taken for a proof of the successful insufflation.

The beneficial effect of the treatment was noticed within one week by a decrease of the attacks in violence and frequency. The time required for a complete cure varied from one to four weeks; in general older children and adults were cured more quickly than young children. And the writer thinks that the time necessary for a cure could perhaps be essentially shortened by more frequent insufflations of smaller doses and by improving upon the *modus operandi*.

2. *Treatment of Diphtheria.* GIBBONS. (*Pacific Med. and Surg. Jour.*, January, 1877.)

The most valuable agent in the materia medica for the treatment of diphtheria is tinct. ferri chloridi. The usual dose of five or ten drops every two hours is too small to yield good results; but it should be given so that an adult patient in twenty-four hours will get from ℥ ss. to ℥ i. Smaller doses do not exert a specific action on the disease. If the stomach gives way, and the medicine is rejected, the tartrate of potassa might be substituted. Usually twenty-four hours of the iron treatment will suffice.

W. F. L.

3. *On the Principles of Antipyretic Action.* MAY. (*New York Med. Jour.*, February, 1877.)

Of the physiological action of cold, Dr. M. says: "When the animal body is exposed to cold, a sudden loss of heat by radiation takes place, and the process of chemical change is retarded; and if the cold is sufficiently intense, a complete cessation of these chemical changes follows"—showing the therapeutic indications of cold in the treatment of fevers; that to lower the temperature, and check the excessive chemical changes, cold must be applied. Of quinine, he thinks that its antipyretic properties are beyond dispute. The fact that it has power to preserve milk, flesh, wine, etc.; of arresting alcoholic fermentation, and preventing putrefaction; that it destroys all kinds of infusorial life, are sufficient proof of its antipyretic power.

Dr. M. believes that sunstroke can be successfully treated by subcutaneous injections of quinine, in proof of which he quotes from cases reported by Surgeon-Major Hall, who had treated quite a number successfully, by dissolving five grains of quinine in five minims of dilute sulphuric acid and fifty minims of water, and injecting in different places about the shoulders.

The best antipyretic results are obtained by administering large doses of the drug when the temperature is high, and small ones when the heat is less.

Alcohol is another remedy which exerts its antipyretic action by supplying force to the body; such force being needed to build up the system when under the influence of fever poison. The author gives a number of experimental cases on dogs, by Dr. Anstie, showing that "the average human adult is capable of decomposing 1½ ounces of pure alcohol"; however, the amount oxidized in the body depends entirely upon the condition of the system at the time taken. Care must be exercised not to give a larger quantity than the system is capable of oxidizing, else its narcotic effects, such as flushing of the face, giddiness, etc., may do harm. W.F.L.

4. *On a Mode of Generating Sulphurous Acid for use as a Disinfectant.* KEATES. (*Lancet.*)

Bisulphide of carbon is a compound of one atom of carbon with two atoms of sulphur ($C. S._2$); it is a dense, mobile liquid, heavier than water, and intensely inflammable, burning in the air like spirit of wine. During combustion the constituents of the bisulphide combine with the oxygen of the air, producing sulphurous and carbonic acid gasses; but as one hundred parts contain by weight as much as eighty-four parts of sulphur, which will give, in burning, one hundred and sixty-eight parts of sulphurous acid, it will be seen that the volume of this gas from a given quantity of bisulphide greatly exceeds that of the carbonic acid, and is comparatively very large.

The bisulphide of carbon can be burned in a common spirit lamp, and by a modification of the method of burning, the amount of sulphurous acid produced in a given time can be regulated to any given extent.

It is a property of the bisulphide of carbon to dissolve in fat, oils and hydrocarbon liquids, such as petroleum; so by mixing it with any one of these liquids, and burning the mixture in a properly constructed oil or petroleum lamp, sulphurous acid will be generated with the other usual products of the combustion of such materials. As the sulphurous gas is generated *pari passu* during the combustion of the bisulphide, it diffuses itself in the air, which in a short time will become completely impregnated with it.

Sulphurous acid generated in this way can be applied with facility to the disinfection of any place or object. It must be observed that the bisulphide of carbon is extremely volatile, having its boiling point as low as 102° F. It is therefore necessary that the lamp in which it is burned should be furnished with a well fitting screw cap, to prevent the liquid from evaporating, and at the same time to keep its peculiar odor from escaping. J. S. K.

5. *Anhidrotics.* FOTHERGILL. (*Practitioner*, December, 1876.)

Anhidrotics are agents which check profuse sweating. They are administered internally or applied externally. Of the latter, mineral or vegetable acids are the most valuable. Of the remedies administered internally, the chief are, dilute phosphoric acid; other acids; astringents, mineral and vegetable; oxides, as of silver and zinc; tonics, as quinine; and some members of the solanaceæ, as belladonna and hyoscyamus.

The most potent of all anhidrotics, in the experience of Dr. F., is belladonna. For the arrest of the exhausting night perspirations of phthisis, belladonna is as potent as digitalis is in giving tone to a feeble heart.

To produce these effects it is necessary to use from the 1-75 to the 1-25 gr. atropia by the mouth, the latter dose rarely failing. After the effect is produced, much smaller doses will maintain it. If the smaller dose is commenced with at first, and by gradual increase the larger dose at last reached, Dr. F. has never seen any alarming symptoms of poisoning, since belladonna produces marked toxic effects before a fatal dose is reached.

J. S. K.

6. *The Practical Uses of Bromide of Camphor.* (*Lancet; Practitioner*, December, 1876.)

In regard to the physiological action of bromide of camphor, it may be stated that it diminishes the number of the beats of the heart, causes contraction of the smaller blood vessels, lowers the temperature, and subsequently induces a more or less marked tendency to sleep. It has been employed by Deneffi, O'Hara and Berger in delirium tremens. The first two advocate its employment as beneficial, while Berger makes some reservations. In insomnia, and especially in that form of sleeplessness which is connected with heart lesions or cerebral hyperæmia, Bournville, Lawson and Pathault declare they have observed encouraging results. Riemer and Hammond state that bromide of camphor proved of use in spasms or convulsions brought on by teething. A great many observers have employed it in epilepsy; and whilst some of them think they have seen the epileptic fits become more and more rare under the influence of the drug, others have only noted a remarkable diminution of vertigo (or *petit mal*) following its use. Chorea and hysteria are, however, the nervous disorders in which bromide of camphor has been most extensively used, and with the best effects.

Turning to another class of disease—namely, affections of the urino-genital organs—it appears that in a case of anal and vesical tenesmus, dependent on peri-uterine phlegmon, Dr. Siredey, of Lariboisière Hospital, has recorded the successful employment of the bromide. In cystitis of the neck of the bladder, again, Dr. Lannelongne states that the beneficial action of the bromide is particularly observable when the cystitis is painful and the pain is not dependent on any organic lesion (neuralgic cystitis); 2, in cystitis of the neck due to congestion; and, 3, when catarrh is mild and when acute prostatitis is added to inflammation of the neck of the bladder.

In a case of symptomatic priapism recorded by Dr. Longuet, bromide of camphor was employed with success; and Dr. Petrovitz has recorded some interesting cases of gonorrhœa in which painful erections were stopped by the use of the capsules of bromide of camphor. J. S. K.

7. *A Ready Solvent for Salicylic Acid.* DUFFEY. (*Br. Med. Journal.*)

Dr. D. states that a permanently clear solution of salicylic acid can be conveniently obtained by dissolving it in liquor ammoniæ acetatis. This solution is more palatable than any hitherto advised, and is less apt to cause the burning sensation in the throat and gastric irritation which often attend the administration of salicylic acid in large doses.

Formula:

℞ Acidi Salicylici .................................... gr. cxx.
Liq. Ammon. Acetatis .................................... ℥ ij.
Aquæ .................................... ℥ vj.
M. Ft. mistura.

One-eighth part contains fifteen grs. J. S. K.

